# Increased Prevalence of Colonic Polyps in Patients with Ampullary Adenoma or Carcinoma: A Single-Center Retrospective Study

**DOI:** 10.3390/jcm15041521

**Published:** 2026-02-14

**Authors:** Muhammed Mustafa İnce, Öykü Tayfur Yürekli, Abdurrahim Yıldırım, Hayriye Tatlı Doğan, Osman Ersoy

**Affiliations:** 1 Department of Gastroenterology, University of Health Sciences, Ankara Bilkent City Hospital, 06800 Ankara, Türkiye; 2 Department of Gastroenterology, Faculty of Medicine, Ankara Yıldırım Beyazıt University, 06800 Ankara, Türkiye; oykutayfur78@yahoo.com (Ö.T.Y.); dr.abdurrahim@hotmail.com (A.Y.); oersoy@yahoo.com.tr (O.E.); 3 Department of Pathology, Faculty of Medicine, Ankara Yıldırım Beyazıt University, 06800 Ankara, Türkiye; hayriyetatli@gmail.com

**Keywords:** colonic polyps, ampulla of Vater, duodenal neoplasms, colonoscopy

## Abstract

**Background/Objectives**: Ampullary adenomas are neoplasms of the gastrointestinal tract with malignant potential. They are thought to develop through pathways similar to those involved in colorectal neoplasia. This study aimed to determine the prevalence of colonic polyps in patients with ampullary adenoma. **Methods**: This retrospective study included a total of 35 patients with ampullary adenoma diagnosed between 2023 and 2024 and 105 sex-matched controls. Colonoscopic findings of the patient and control groups were compared with respect to polyp prevalence. In addition, the effects of dysplasia grade of the ampullary adenoma and patient age on polyp prevalence were evaluated. **Results**: The study included 35 patients (57% male) and 105 controls (59% male). The mean age was 67.06 ± 13.32 years in patients and 61.28 ± 8.42 years in controls. Colonic polyps were detected in 13 (57%) patients in the low-grade dysplasia (LGD) group, 6 (66%) patients in the high grade dysplasia (HGD) or adenocarcinoma group, and 54 (51%) patients in the control group (*p* = 0.02). After adjusting for age, colonic polyps remained significantly more frequent in the adenoma group than in controls (*p* = 0.05). Polyp prevalence was not associated with dysplasia grade on ampullary biopsy, and no significant differences were observed between groups regarding polyp histopathology, location, or size. **Conclusions**: In conclusion, our study indicates that colorectal polyp prevalence is increased among patients with ampullary adenomas and that this association may be independent of age as well as dysplasia severity. Therefore, colonoscopic evaluation may be recommended for all patients diagnosed with ampullary adenoma.

## 1. Introduction

Ampullary adenomas are mostly benign neoplasms originating from the distal union of the biliary duct and pancreatic duct. They represent approximately 6–17% of periampullary neoplasms [1]. Autopsy series revealed an incidence of 0.04–0.12% [2]. Despite the mostly benign nature of these neoplasms, they carry a real potential of malignant transformation, causing a significant clinical impact. Some studies report that up to half of lesions may harbor carcinoma foci [3]. Recently, an increasing number of endoscopies and radiologic studies has caused an increased incidence of ampullary adenomas. The first treatment choice is always endoscopic papillectomy due to its less invasive nature, and these patients undergo surveillance programs for the detection of recurrence [4].

Ampullary and colonic cancers are two intestinal malignancies with presumably similar pathways progressing from adenoma to carcinoma. Previous research has demonstrated that the occurrence of one of these cancers is associated with an elevated risk of developing the other cancer type. A US study showed that in patients diagnosed with ampullary cancer, there was an increased risk of colon cancer compared to the normal population [5]. In patients with hereditary polyposis syndromes such as familial adenomatous polyposis (FAP) and hereditary non-polyposis colorectal cancer (HNPCC, Lynch syndrome), the incidence of ampullary neoplasms appears to be higher than in the general population. Moreover, some studies suggest a markedly increased risk of ampullary carcinoma, particularly among patients with FAP [6].

We aimed to compare the colonoscopy results of patients with ampullary adenoma or carcinoma detected upon endoscopic retrograde cholangiopancreatography (ERCP) with a control group of patients over age 50 undergoing colonoscopy for colon cancer surveillance. After seeing the adenomatous appearance of an ampulla with a side-viewing endoscope, pathologic confirmation was needed for inclusion in this study. By this comparison, we aimed to determine whether patients with ampullary adenomas carry a higher risk of colon cancer compared to the healthy population.

## 2. Materials and Methods

### 2.1. Study Design

We included patients with endoscopic suspicion of ampullary adenoma or carcinoma during ERCP performed between 2023 and 2024, with histopathologic confirmation on ampullary biopsy. Patients with a known history of periampullary or duodenal neoplasia and those younger than 18 years of age were not included in the study. Patients without histopathological evidence of dysplasia and those who did not undergo colonoscopy during the same period were excluded. The control group was chosen as patients applying to gastroenterology polyclinics and undergoing colonoscopy procedures for the purpose of colon cancer surveillance. Patients with known FAP or precancerous lesion of the gastrointestinal tract and patients with inflammatory bowel disease were excluded from the study.

Colonoscopy findings for patients and control groups were analyzed. After recording the demographic findings of the groups, lesions identified through colonoscopy were classified according to localization, size and histologic findings. In addition, precancerous polyps detected through colonoscopy were classified according to their degree of dysplasia and compared between groups. Histopathologic findings of ampullary adenomas and adenomatous lesions of the colon were classified as low-grade dysplasia (LGD), high-grade dysplasia (HGD) and carcinoma based on the Vienna classification [7].

Ampullary pathologies of the patient group were analyzed by dividing the patients into two groups. The first group was defined as patients having LGD and the other group was defined as patients having HGD or carcinoma. Colon polyps were classified according to polyp size (<1 cm or >1 cm) and polyp localization (right colon, transverse colon, left colon or multiple localizations). In patients with more than one polyp, if there was a polyp > 1 cm in size, the localization of this polyp was taken for statistical analysis, but if all the polyps were <1 cm, then the patient was grouped as having multiple localizations.

Examination of the ampulla using a duodenoscope and biopsy sampling was performed by a single operator (O.E). Colonoscopy procedure was performed by a single endoscopist (Ö.T.Y). Biopsy specimens were evaluated by a single pathologist with significant expertise on gastrointestinal system pathology (H.T.D). This was a single-center, retrospective case–control study.

### 2.2. Statistical Analysis

The data were analyzed using SPSS version 25. Median, mean, and standard deviation (SD) values were calculated and presented in the corresponding tables. Frequency values obtained in the patient and control groups were compared using the chi-square test. After controlling for the effects of certain demographic variables, the impact of ampullary adenoma or carcinoma on the likelihood of patients having polyps was examined through ordinal regression analysis. The significance level of the 95% confidence interval was used for intergroup comparisons, and *p*-values less than 0.05 were considered statistically significant.

## 3. Results

**Figure 1:**
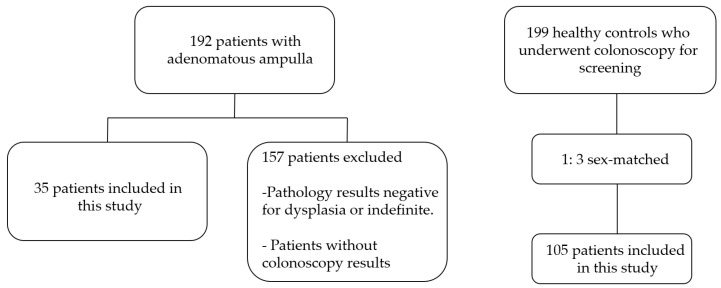
The flow chart of the study.

A total of 192 patients with biopsies taken from adenomatous-appearing ampulla with a side-viewing endoscope between 2023 and 2024 were evaluated for inclusion. A total of 157 patients with negative or indefinite dysplasia pathologies and patients who did not undergo colonoscopy were excluded. A total of 199 patients on whom colonoscopy was performed for colon cancer surveillance during the same period were evaluated for the control group. In order to control for the factor of gender, 105 patients with a ratio of 1:3 were included in the control group (Figure 1).

**Figure 2:**
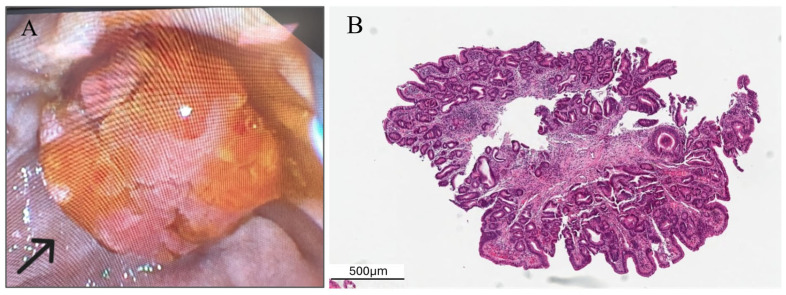
One patient diagnosed with adenoma of the ampulla of Vater. (**A**) The endoscopic view of the adenoma of the papilla of Vater (black arrow). (**B**) Microscopic view of a tubular adenoma of the ampulla with low-grade dysplasia.

The patient group was composed of 35 patients, 20 males (57%) and 15 females (43%). The control group was chosen as 105 patients, 62 males (59%) and 43 females (41%). The mean age of the patients was 67.06 ± 13.32, while it was 61.28 ± 8.42 in the control group. Three patients had a history of colon resection for colon cancer in the patient group. For this reason, these patients were not assessed for polyp frequency. ERCP was performed in 11 patients for cholangitis, 11 patients for choledocholithiasis, 1 patient for biliary leakage, and 12 patients for distal biliary stricture (Figure 2). Among patients who underwent ERCP for cholangitis, 7 were subsequently diagnosed with ampullary carcinoma and 4 with pancreatic cancer. Colonic polyps were detected in 13 (57%) patients in the LGD group, 6 (66%) patients in the HGD or adenocarcinoma group, and 54 (51%) patients in the control group (Table 1).

A significantly higher prevalence of colonic polyps on colonoscopy was demonstrated in patients with ampullary adenoma compared with the control group, based on ampullary biopsy findings (Chi-Square = 9.79; *p* = 0.02) (Table 2). After controlling for the effect of age, the frequency of colonic polyps remained significantly higher in the ampullary adenoma and carcinoma group compared with the control group (*p* = 0.05).

The most frequent type of polyp was tubular adenoma, both in the patient and the control groups. Two patients were diagnosed with colon cancer in the control group (Table 1). There were no significant differences regarding polyp type between the groups (Chi-Square = 0.69; *p* = 0.71) (Table 1).

There was a tendency for left-sided localization and multiple polyps in the patient group. Likewise, polyps were more commonly localized in the left colon in the control group. In both groups, polyps were mostly <1 cm in size (Table 1). There were no significant differences regarding polyp localization (Chi-Square = 1.91; *p* = 0.59) and polyp size between the adenoma and the control groups (Chi-Square = 1.65; *p* = 0.20) (Table 1).

No significant differences (*p* > 0.05) existed between the low-grade and control groups regarding the distribution of polyp locations (right, transverse, left, or multiple). The low-grade group showed a slightly higher prevalence of left-colon localization and multiple lesions; however, this trend was not statistically significant. Larger polyps (>1 cm) appeared to be somewhat more frequent in proportion in the low-grade group (27% vs. 15%), but the difference was not significant (Fisher *p* ≈ 0.39). The pattern of pathology types was very similar between low-grade and control groups. Due to a small sample size in the low-grade group, Fisher’s exact test was performed. The results supported the results mentioned above. The odds of neoplastic lesions were slightly higher in the low-grade group, but this difference was not statistically significant. Low-grade cases had about twice the odds of having a large (>1 cm) polyp, but again, the difference was not significant. Low-grade lesions were somewhat less likely to occur distally, though this pattern was not statistically meaningful (Table 3).

The χ^2^ tests indicated no significant differences across any of the tables (e.g., Pearson χ^2^ = 0.913, *p* = 0.822 for polyp location; χ^2^ = 0.548, *p* = 0.760 for polyp pathology; χ^2^ = 1.215, *p* = 0.270 for polyp size). Consequently, based on the existing data, there was no statistically significant difference between the high-grade group and the control group regarding location, size, or pathology. Moreover, symmetric measures (Pearson’s R/Spearman) approximated zero, indicating an absence of a significant relationship between the variables. Due to a small sample size in the high-grade group, Fisher’s exact test was performed. The results supported the results mentioned above. The high-grade group had about 2.7 times more large polyps (more than 1 cm) than the control group, but the difference was not statistically significant (Table 4).

## 4. Discussion

The frequency of sporadic duodenal adenoma detection increases with the increasing number of upper gastrointestinal endoscopies. Early resection of these lesions decreases the risk of developing invasive cancers, but there is not enough data about the natural course of duodenal adenomas detected in patients without polyposis syndromes and the frequency of concurrent colonic polyps, and so an appropriate surveillance program was not put forward for these patients.

There are studies in the literature that have examined the association between duodenal or ampullary adenomas and colorectal polyps using various methodological approaches. Some of these previous studies have shown that the frequency of colorectal polyps increased in patients with duodenal (ampullary and non-ampullary) adenomas [8,9,10]. There are studies that included sporadically detected adenoma patients during endoscopy due to different indications. We only included ampullar adenoma or carcinoma detected during the ERCP procedure and did not include non-ampullary adenoma patients. In this way, we tried to analyze a more specific and homogeneous patient cohort and obtained data relevant to these patients.

In previously reported studies, patients with duodenal adenomas were predominantly male [8,9,10]. Similarly, in the current study, the majority of adenoma patients were male (57%). Recent studies have demonstrated that the detection rate of polyps during screening colonoscopy is higher in males [11,12]. The male predominance of both duodenal and colorectal neoplasms, along with their tendency to coexist, supports the hypothesis that these conditions may develop through similar pathways.

Scheneider et al. compared 26 ampullary adenoma patients with 104 asymptomatic controls and found no significant difference in the incidence of colorectal polyps (23% vs. 26%) [13]. On the other hand, Zhou et al., in a similar study, compared 95 ampullary adenoma patients with 380 asymptomatic controls and found an increased incidence of colorectal polyps (60% vs 34%) [12]. Similarly we found an increased incidence of colorectal polyps in the adenoma group (54% vs. 51%). Furthermore regression analyses revealed that polyp incidence is higher in ampullary adenoma patients even after controlling for the factor of age. Given that age is considered an important factor in the development of colorectal polyps, our study demonstrated that polyp prevalence increased in the adenoma group independently of age.

In two recent studies conducted in our country, the polyp detection rates during screening colonoscopy were reported as 30.9% and 34.1%, respectively [14,15]. In the current study, a higher rate was observed in the control group (51%). This relatively high polyp detection rate during screening colonoscopies may not reflect the true prevalence in the general population and should be considered a limitation of the study. However, this finding may be associated with our institution’s role as a tertiary referral hospital and the extensive experience of the endoscopy team, which has more than 20 years of clinical practice. Additionally, the institutional approach of removing all detected polyps regardless of size may have contributed to the higher detection rates observed.

A previous study revealed that colorectal polyps were mostly located in the right colon in patients with duodenal adenomas [16]. But more recent studies found no difference regarding polyp localization between patients and control groups [17,18]. In our study, we found most colorectal polyps were located in the left colon or there were multiple in number, although these differences did not reach statistical significance. This finding is interpreted as indicating that the association between ampullary neoplasia and colorectal polyps represents a general increase in risk rather than one confined to a specific colonic segment.

Further subgroup analyses were done to define the relationship between the degree of dysplasia and the incidence of colorectal polyps. Patients with low-grade dysplasia of ampullary adenoma were compared with patients with HGD or carcinoma. No significant difference was observed between the two groups with respect to the presence of polyps. However, in both subgroups, the frequency of polyps was significantly higher than in the control group. Although no difference was detected between the groups in terms of high-risk lesions or advanced colorectal neoplasia, an increased overall polyp prevalence was observed in patients with ampullary adenoma. Therefore, rather than recommending routine surveillance colonoscopy, our findings suggest that patients with ampullary adenoma may be considered for individualized assessment regarding colorectal screening, based on their overall clinical risk profile. To the best of our knowledge, our study is the first in the literature to investigate the prevalence of colorectal polyps according to the degree of dysplasia in ampullary adenomas. However, given the limited number of patients in our study, further investigations with larger patient numbers would be valuable to better clarify the relationship between dysplasia grade and colonic polyp prevalence.

The most important limitation of this study is the limited number of patients included. This is mainly due to the limited number of ampullary adenoma patients. This limited cohort reduces the statistical power and warrants cautious interpretation of statistically significant findings. Age of the control group could not be matched with the patient group due to the increased age of ampullary adenoma patients and the fact that surveillance colonoscopies are not routinely warranted in older ages. We attempted to mitigate this limitation by using regression analysis to adjust for age differences. Although the association between ampullary adenoma and colonic polyp prevalence remained statistically significant after adjustment, residual confounding cannot be excluded. Multicenter studies can reach an increased number of ampullary adenoma patients to define more conclusive results. Another limitation is that we did not do propensity score matching when choosing the control group, but we tried to manually select the best-matching control group individuals according to the age and sex of the patients. Finally, although no specific colonoscopic examination protocol was applied to the study patients, the endoscopist performing the procedures was not blinded to the presence of ampullary adenoma, which represents a potential source of detection bias.

In the current study, examination with narrow band imaging (NBI) could not be performed due to technical limitations. Consequently, we were unable to assess the pit patterns of the lesions or compare these findings with the biopsy results.

In a previous study, gastric cancer or atrophic gastritis was more prevalent in neoplasms located on the oral side of the major papilla in patients with sporadic non-ampullary duodenal adenomas, whereas colorectal adenoma or carcinoma was more prevalent in neoplasms located on the anal side. The association with gastric pathologies was attributed to increased gastric acid secretion secondary to Helicobacter pylori infection, leading to a more acidic environment in the proximal duodenum. In contrast, the association with colorectal pathologies was thought to be related to a possible increase in bile acid levels [19]. In the present study, upper gastrointestinal endoscopy was not performed in either the patient or control groups, and biopsies obtained from the ampulla were not directionally oriented. Therefore, the association between ampullary adenomas and gastric pathologies could not be evaluated.

The most important strength of this study was the fact that both the ERCP and colonoscopy procedures were done by a single endoscopist, thereby limiting interobserver variation in the detection rate of polyps. Furthermore, the assessment of all biopsy specimens by a single pathologist with expertise in gastrointestinal pathology minimized observer-related bias in the microscopic characterization of the lesions.

## 5. Conclusions

In conclusion, our study indicates that colorectal polyp prevalence is increased among patients with ampullary adenomas and that this association may be independent of age as well as dysplasia severity. An individualized approach to colorectal evaluation in patients diagnosed with ampullary adenoma may be considered, taking into account overall clinical risk factors. Further studies are needed to establish a standardized colonoscopy surveillance protocol specific to these patients.

## Figures and Tables

**Table 1:** Distribution of gender, age and polyp characteristics between the study groups.

	Patients (*n* = 35)	Controls (*n* = 105)
Sex (male)	20 (57%)	62 (59%)
Age (mean ± SD)	67.06 ± 13.32	61.28 ± 8.42
ERCP Indications Cholangitis Choledocholithiasis Biliary leakage Distal biliary stricture	11 (31%) 11 (31%) 1 (3%) 12 (35%)	
Polyp frequency LGD HGD + Adenocarcinoma	13 (57%) 6 (66%)	54 (51%)
Polyp pathology Hyperplastic polyp Tubuler adenoma Adenocarcinoma	4 (21%) 15 (79%) 0	12 (22%) 40 (74%) 2 (3%)
Polyp localization Right colon Transvers colon Left colon Multiple localization	1 (5%) 4 (21%) 7 (37%) 7 (37%)	10 (19%) 9 (17%) 21 (40%) 12 (23%)
Polyp size Smaller than 1 cm Bigger than 1 cm	14 (74%) 5 (26%)	44 (81%) 8 (19%)

ERCP: endoscopic retrograde cholangiopancreatography, SD: standard deviation, LGD: low-grade dysplasia, HGD: high-grade dysplasia.

**Table 2:** Colonic polyp frequency according to ampullary biopsy results.

Colonoscopy	Tubular Adenoma-LGD	Tubular Adenoma-HGD + Adenocarcinoma	Control
No polyp	10	3	51
There is polyp	13	6	54
Total	23	9	105

LGD: low-grade dysplasia, HGD: high-grade dysplasia.

**Table 3:** Odds ratios for polyp characteristics in low-grade dysplasia versus controls.

Variable	Odds Ratio (OR)	Fisher *p*-Value	CI
Pathology (neoplastic vs. non)	1.43	1.000	[−0.90–1.83]
Size (>1 cm vs. ≤1 cm)	2.06	0.389	[−0.42–1.23]
Localization (distal vs. proximal)	0.39	0.310	[−0.37–1.22]

CI: confidence interval.

**Table 4:** Odds ratios for polyp characteristics in high-grade dysplasia versus controls.

Variable	Odds Ratio (OR)	Fisher *p*-Value	CI
Pathology (neoplastic vs. non-neoplastic)	0.57	0.617	[0.10–0.50]
Size (>1 cm vs. ≤1 cm)	2.75	0.274	[0.50–5.50]

CI: confidence interval.

## Data Availability

The data presented in this study are available on request from the corresponding author due to ethical reasons.
